# Biomedical Applications of Chinese Herb-Synthesized Silver Nanoparticles by Phytonanotechnology

**DOI:** 10.3390/nano11102757

**Published:** 2021-10-18

**Authors:** Rehmat Islam, Leming Sun, Lianbing Zhang

**Affiliations:** Key Laboratory of Space Bioscience and Biotechnology, School of Life Sciences, Northwestern Polytechnical University, Xi’an 710072, China; rehmatislam@mail.nwpu.edu.cn (R.I.); lbzhang@nwpu.edu.cn (L.Z.)

**Keywords:** Chinese herbal, silver nanoparticles, phytonanotechnology, bioactive molecules, biomedical applications

## Abstract

Recent advances in nanotechnology have opened up new avenues for the controlled synthesis of nanoparticles for biomedical and pharmaceutical applications. Chinese herbal medicine is a natural gift to humanity, and it has long been used as an antibacterial and anticancer agent. This study will highlight recent developments in the phytonanotechnological synthesis of Chinese herbal medicines to utilize their bioactive components in biomedical and therapeutic applications. Biologically synthesized silver nanoparticles (AgNPs) have emerged as a promising alternative to chemical and physical approaches for various biomedical applications. The comprehensive rationale of combinational or synergistic effects of Chinese herb-based AgNPs synthesis was investigated with superior physicochemical and biological properties, and their biomedical applications, including antimicrobial and anticancer activity and wound healing properties. AgNPs can damage the cell ultrastructure by triggering apoptosis, which includes the formation of reactive oxygen species (ROS), DNA disintegration, protein inactivation, and the regulation of various signaling pathways. However, the anticancer mechanism of Chinese herbal medicine-based AgNPs is more complicated due to the potential toxicity of AgNPs. Further in-depth studies are required to address Chinese herbs’ various bioactive components and AgNPs as a synergistic approach to combat antimicrobial resistance, therapeutic efficiency of drug delivery, and control and prevention of newly emerged diseases.

## 1. Introduction

The emergence of nanotechnology has gained more attention, with effective applications appearing in recent years, including biomedical, drug delivery, textiles, agriculture, food industry, cosmetics, and electronics applications [1,2,3,4,5,6,7,8,9]. Metal nanoparticles (MNPs), including gold, iron, zinc and silver nanoparticles, have been extensively investigated in the last decade [10,11,12,13,14,15]. Among them, silver nanoparticles (AgNPs) have emerged as prominent antimicrobial agents due to their unique physicochemical characteristics, chemical stability, and therapeutic, biomolecular detection, and preservative properties [16,17,18]. Silver is mainly used as a sanitizer or disinfectant in topical ointment creams to heal wounds and restrict bacterial growth. AgNPs appeared as new potential antibacterial agents to replace silver ions. The inactivation of silver caused by its complex formation or precipitation has comparatively limited its effects and retains only for a short time [19]. However, researchers are looking for new therapeutic strategies to deal with microbial infections.

Numerous chemical and physical approaches have been utilized for the preparation of AgNPs. For instance, the chemical reduction method is widely used, particularly the Creighton method, to obtain desired monodispersed and small-size nanoparticles using various chemical additives [20]. The primary components used in this method are (i) metal precursor, (ii) a reducing agent, and (iii) a stabilizing agent. The chemical synthesis method using toxic chemicals may limit their medical applications and harm the environment by producing hazardous byproducts [21]. Physical synthesis approaches are also used without chemical additives, such as mechanical ball milling, laser ablation, and vapor-based approaches [22,23,24]. Sputtering and evaporation are prominent physical vapor-based deposition approaches, which involve the bombardment of the target material, which condenses the sputter of atoms on the substrate. Physically synthesized AgNPs may have a stable and uniform average size with high purity. However, the physical method also possesses several drawbacks, including large area settings, high external energy requirements, being time consuming, and requiring sophisticated equipment [25]. The physical method is considered extremely difficult for stabilization to prevent agglomeration and oxidation processes in the absence of stabilizing and capping agents. To overcome the limitations in physical and chemical synthesis approaches, biological synthesis is considered the best alternative option.

Biological synthesis, often known as ‘green synthesis,’ is a new trend in nanomaterial synthesis that offers the advantage of natural resource utilization and a simple, cost-effective, and environmentally friendly approach [26,27,28]. The biological pathway for AgNPs synthesis is dependent on macromolecular compounds found in bacteria, fungi, and algae and plant bioactive components. Plant-mediated AgNPs synthesis has gained popularity due to its strong reducing capability, fast synthesis, non-pathogenic action and antimicrobial activity [29]. Using Chinese herbs, AgNPs have been synthesized from *Panax ginseng Meyer*, *Dendropanax mobifera Leveille*, *Angelica pubescens, Tamarix gallica,* and *Terminalia chebula* [30], showing potential antimicrobial, anticancer, and antioxidant activity [31]. Modern scientific approaches suggested that AgNPs could be used as a potential antimicrobial agent against multi-drug-resistant strains with minimum human toxicity and extensive clinical applications [32,33].

Phytonanotechnology has emerged as a new approach in using Chinese herbal or medicinal plants for pharmaceutical applications. Each plant contains an orchestra of phytochemicals with higher therapeutic values and is free of harmful substances [34,35]. These chemically complex phytochemicals can reduce Ag^+^ to Ag^0^, such as peptides, enzymes, carbohydrates, and various organic compounds that serve best for medical applications. Silver has the potential to restrict bacterial growth by interfering with cellular molecules [36]. The biosynthesis of AgNPs using *Saraca indica* leaf extract is employed to characterize bactericidal efficacy against *Escherichia coli* (*E. coli*), *Staphylococcus aureus* (*S. aureus),* and *Micrococcus luteus* [37], suggesting that medicinal plant-based AgNPs with a spherical shape and desired size of 23 nm have potent antibacterial activity. Similarly, the *Withania coagulans* herb was widely used as a folk remedy against diabetes, hypercholesteremia, and cancer. The leaf extract *W. coagulans* is used for AgNPs synthesis to assess its antibacterial, antioxidant, and cytotoxic properties [38]. Recently, the biological synthesis of AgNPs using *Curcuma longa* aqueous extract to exploit its antibacterial activity against *E. coli* and *Listeria monocytogenes* [39] showed the potential reducing ability of silver salts in comparison to other plants. However, further research is required to analyze the complicated molecular composition of Chinese herbal extracts and their interaction with AgNPs.

Chinese herbal root and leaf extract has been widely used to synthesize AgNPs and render unique antibacterial and antiviral activity. Traditional Chinese medicine (TCM) is being progressively used for clinical purposes in the treatment of different diseases [40,41]. *Cinnamomum cassia*, a Chinese herb, has been reported to be less toxic and exhibit potential antimicrobial activity. It could be formulated with other medically important herbs for multi-purpose use in medical fields [42]. Similarly, the root extract of *Angelica pubescens* Maxim. is widely used to synthesize AgNPs for in vitro antioxidant activity and control bacterial pathogenicity [43]. Moreover, the cytotoxic effect was further analyzed in murine macrophages (RAW264.7) as their anti-inflammatory potential [30]. It is evident from previous research that AgNPs and Chinese herbs are potential antimicrobial agents and have high prospects for clinical purposes. As a result, the combination or synergistic effects of Chinese herbs and AgNPs harnessed antibacterial and healing properties with minor adverse effects, making it the best candidate for biomedical applications. The progression of comprehensive research on Chinese herb-mediated AgNPs via phytonanotechnology has the potential to have enormous future implications.

This study aims to outline the recent development of Chinese herb-based AgNPs as prospective antimicrobial agents and wound-healing agents. The combinational or synergistic effects of Chinese herb-synthesized AgNPs will be discussed with unique physicochemical properties and medical uses, such as antimicrobial, antioxidant, anticancer, and wound-healing properties. Furthermore, the clinical significance of Chinese herbs’ essential bioactive components for AgNPs synthesis and therapeutic effectiveness will be discussed.

## 2. Chinese Herbal Medicine

For millennia, Chinese herbs have been utilized as a pharmaceutical and dietary supplement. Still, most researchers have focused on clinical therapy, despite its properties for nanomaterial synthesis and applications being prominent [44]. Chinese herbal medicine is thought to have been used for more than four thousand years, primarily during the Xia era. Shennong, the divine farmer, is considered to be the inventor of Chinese herbal medicine [45]. These Chinese herbs possess various potentially bioactive molecules such as flavonoids, quinones, lignans, tannins, terpenoids, and other endogenous metabolites that could be used as antioxidants [46]. Moreover, it has been demonstrated that bioactive molecules derived from Chinese herbal medicine have played, and are playing, an important role in curing diseases and boosting immunity [47]. These bioactive molecules exhibit remarkable reducing and stabilizing properties, indicating that they have significant therapeutic potential in preventing and treating various complex and heterogeneous diseases, such as cancer.

### 2.1. Development of Chinese Herbal Medicine

Chinese herbal practitioners have been recording their experiences for thousands of years. Chinese herbal medicine is one of the earliest medicinal remedies that have played an important role in curing many diseases. Most Chinese herbs, such as *Cinnamomum cassia, Ephedra sinica,* and *Zingiber officinale,* are commonly used as vital food supplements and folk medicines [48]. The fundamental theories of TCM were written in the 2nd century and are framed on *Huang Di Nei Jing*, which means ‘prevention before disease occurrence’ and contains many methodologies to control and prevent the spread of diseases and their recurrence after recovery [49]. Li Shizhen wrote a classical Chinese text on Chinese herbal medicine in the 16th century, recording 1892 distinct herbs and 11,096 medicines that control and prevent various diseases [50,51]. Nowadays, traditional herbal treatment for human diseases has been replaced by modern Western medicine and pharmaceuticals. However, Chinese herbal medicine is still widely used in China [52]. In the last 70 years, China has established the structure-based foundation of Chinese herbal medicine universities, hospitals, and research institutes to utilize the Chinese herbs for biomedical applications. Chinese herbal medicine is considered a natural reservoir of active compounds, and many researchers have investigated various phytochemicals, including proteins, amino acids, coumaric acid, aldopentose, calcium oxalate and polysaccharides. The composition of these bioactive molecules validates the synergistic effect against microbial pathogenicity [53,54].

The US Food and Drug Administration has reviewed and approved novel molecular entities for clinical use, with natural products accounting for 25%. According to the previous report [44], Chinese pharmaceuticals rely on traditional herbal medicines up to 30–50%. Chinese herbs and their products have gained popularity in Asia as well as in Western countries. A survey was conducted in 2004 regarding medical treatment preferences of TCM and Western integrative medicines among people [55]. According to the findings, 71% favored integrative medicines, whereas 19% preferred TCM. Another study conducted in 2015 found that 66% of 2712 coronary heart patients were treated by Western medicine, 30% preferred integrative medicine, and only 3.1% favored TCM [56]. Moreover, Chinese herbal medicinal concepts, ‘yin vs. yang’ and ‘hot vs. cold’ definitions, pose safety concerns and do not fit with modern physiology and molecular biology definitions [51,57]. Therefore, the development of new guidelines for Chinese herbal drug administration is urgently required to cope with the upcoming challenges of new emerging diseases.

According to the theory and principles of Chinese herbal medicine compatibility, component-based Chinese medicine is a new type of TCM formulated from herbal extract standard ingredients in fixed proportion [58]. Astragali radix are the dried roots of *Astragalus membranaceus* (Fisch.), one of the most common herbal drugs with potential antioxidant properties and antiviral activities. More than 100 compounds have been identified in Astragali radix, such as flavonoids, saponins, and polysaccharides, and their biological properties were highlighted [59].

### 2.2. Biological Synthesis of Chinese Herbal AgNPs

To date, numerous study reports on the green synthesis of AgNPs using a variety of microorganisms, plant parts, or herbal extract have increased exponentially. Compared to chemical or physical synthesis routes, the biological method is based on a redox reaction, is easy to handle, requires normal temperatures, and minimizes the use of toxic chemicals. In biological synthesis, microorganisms such as bacteria, yeast, fungi, and algae are used to synthesize AgNPs and investigate their antioxidant and reducing properties [60]. However, plants or Chinese herb-mediated synthesis has gained popularity due to its easy availability, non-toxicity, cost-effectiveness, and reducing/capping agent content, such as citric acid, alkaloids, vitamins, flavonoids and phenolic compounds exhibiting strong antimicrobial properties. Aygun reported [61] a novel approach using medicinal plant ethanolic extract *Rheum ribes* for the synthesis of AgNPs and investigated the anticancer and antimicrobial properties. The medicinal plant is enriched with multiple vitamins and polyphenolic compounds with antioxidant properties and helps to reduce and stabilize nanoparticles. From a close perspective, the recent biological synthesis of AgNPs is shifting toward the medical flora, which uses medicinal herbs or plants to reduce and stabilize AgNPs [62]. Fresh, healthy and disease-free parts of Chinese herbs are selected, including leaves, flowers, stems, and roots, and used in the biosynthesis of AgNPs of various sizes and shapes [63]. These extracts contain major bioactive components such as enzymes, alcohol, flavonoids, quinines, terpenoids, and other phenolic compounds. However, understanding the complete biomolecular composition of herbal extracts is a major challenge, as the biochemical composition of these molecules might change substantially between species or even tissues [64].

**Figure 1:**
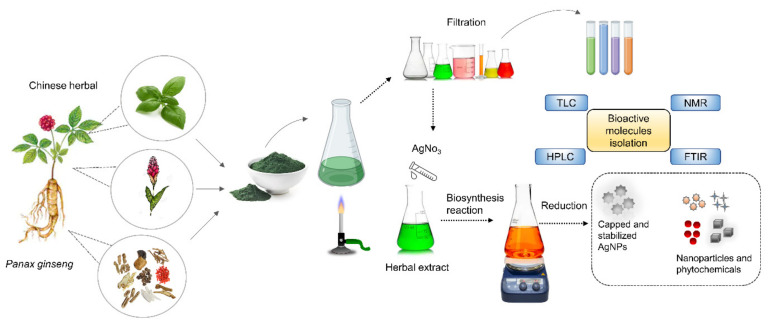
Biological synthesis of silver nanoparticles (AgNPs) from Chinese herbs containing bioactive molecules.

Among biological methods, phytonanotechnological synthesis is considered as a novel approach. The phytochemical reduction method is comparatively slower than a conventional chemical reduction in earlier times. However, this limitation is resolved by using microwave-assisted synthetic techniques so that biological synthesis can be carried out rapidly with good yield [65]. Bioactive components or phytochemicals with various functional groups, such as hydroxyl, carbonyl, and amidogen groups, are ideal for adsorption on the metal surface to reduce Ag^+^ to Ag^0^. Chinese herbal extracts contain various bioactive molecules (carbohydrates, phenolic acids, flavonoids, aminoacids, and proteins). Therefore, they can exhibit multiple functionalities (reducing and stabilizing agents) and acquire different morphological structures with multifunctional organic molecular assemblies. In general, phytochemical-based AgNP synthesis involves three steps: (1) selection or use of herbal extract, (2) study nucleation and growth of AgNPs, and (3) selection of solvent media for extraction of phytochemicals [66], as shown in Figure 1. Recent research has pointed out that, in addition to the quality of herbal extract or metabolites and their concentrations, various other parameters, such as temperature, reaction time, pH, the concentration of metal precursor, and electrochemical potential, can affect the reduction process [67,68]. Therefore, it is easy to obtain the desired size of AgNPs by changing the synthesis parameters.

Despite the multiple benefits of green synthesis of AgNPs using herbal phytochemicals, nanoparticle polydispersity remains an open challenge in various biological systems and requires optimization. Therefore, researchers are interested in developing a reliable method for synthesizing nanoparticles that are homogeneous in size and morphology. Numerous phytocompounds derived from TCM or Chinese herbs alone have been examined for their antimicrobial properties. For instance, at least 289 saponins (Ginsenosides) have been identified in Ginseng or *Panax* species. Over 136 bioactive molecules have been isolated from bulbs of *Allium macrostemon* and *Allium chinense*, including 55 volatile oils, nine nitrogenous compounds, 61 steroidal saponins and 11 others [69]. Numerous Chinese herbs, including *Ephedra sinica*, *Artemisia annua*, *Alpinia officinarum*, *Angelica sinesis*, *Arctium lappa*, *Astragalus membranaceus*, *Chrysanthemum morifolium*, *Lycium chinense,* and *Salvia miltiorrhiza* have antibacterial and antifungal activity [70]. These phytochemicals can significantly alter the size and shape properties of AgNPs. Recently, ethanol extract of *Allium cepa* peel was utilized to fabricate AgNP synthesis, and bioactive compounds were detected on the surface of spherical-shape AgNPs ranging in size from 20 to 50 nm. The size of the biosynthesized AgNPs was influenced by the extract and AgNO_3_ concentration_,_ pH, incubation time, and chemical composition of these compounds [71]. Similarly, phytochemical analysis of Chinese herbal *Ginkgo biloba* revealed that lactone, flavonoids, and polyphenols comprising a variety of functional groups, such as phenolic hydroxyl, carboxyl, and ketone groups, bind to the silver surface. Several of these functional groups engage in reducing silver ions, while some can form complexes with silver ions and still others regulate the size and morphology of resulting AgNPs [72]. FTIR spectra of herbal extract before and after bioreduction showed a shifting peak at 3557.98–3428 cm^−1^ (due to N-H stretching, amides) and C-N stretching mode of aromatic amine group at 1446.61–1379.97 cm^−1^ bands, indicating the involvement of amides, amino groups and polyphenols in the synthesis and stabilization of AgNPs (10–16 nm).

Eun et al. studied methanol extract of *Carpesium cernuum* for AgNP synthesis to determine the relationship between bioactive molecules and AgNP structures [73]. The reduction reaction was evaluated for its effects on yield, colloidal stability, size, and shape. The highest frequently observed size was 12.5–15 nm, which accounts for 26% of the total samples, while the hydrodynamic size increased up to 110.2 nm with a polydispersity index of 0.241. They concluded that phytochemicals and their functional hydroxyl and carbonyl groups residing on the surface of nanoparticles increases the hydrodynamic size. Colloidal stability on the shelf for 28 days showed no significant change in structure and shape, implying that phytochemicals played a key role as a stabilizing agent. To provide new insights, Lü et al. investigated the bioactive compounds in *Gardenia jasminoides* responsible for AgNP synthesis and stability [74]. They revealed that AgNP synthesis is due to reducing aqueous AgNO_3_ by saccharides, carbonyl compounds, or phenolic hydroxyl group, with aldehyde ketones acting as protective groups. Notably, bovine albumin, rutin, gallic acid, and chlorogenic acid demonstrate unique capping capacity. To our understanding, the plethora of Chinese herbal bioactive compounds or molecules are responsible for the synthesis of AgNPs because they function as reducing and capping agents, preventing agglomeration of nanoparticles and allowing better size control and structural stability.

For the preparation of AgNPs, Chinese herbal desired parts are selected and washed repeatedly with Milli-Q deionized water to remove surface contamination and related impurities. After cleaning, the herbal parts are milled into powder and mixed with distilled water and silver nitrate (AgNO_3_) in desired concentrations. Finally, the herbal extract is boiled at 80 to 100 °C on the magnetic stirrer heating pad or in a high-pressure reactor. The color change of the reaction mixture predicts the synthesis of AgNPs [75]. Previously, 33 Chinese herbs collected from the Hong Kong store were extracted using the hydrothermal method, and their bioactivity was tested against antibiotic-resistant bacterial pathogens [76]. Initially, the desired parts of the herbs were soaked for one hour and then boiled repeatedly with distilled water or absolute ethanol (50% or 90%) for two hours. Later, the aqueous or ethanolic extract was processed for filtration and then lyophilized into powder. Over the past ten years, most studies have relied on the biogenic synthesis of AgNPs using plant aqueous extract. Apart from the hydrothermal method, the microwave-assisted method also refers to a rapid heating process by microwave irradiation of silver precursor [77]. In this method, plants or herbal extracts of fruits, leaves, or roots are mixed with AgNO_3_ solution and placed in a microwave oven at a high temperature for a short time.

After cooling the solution, the color change from light yellowish to reddish-brown represents the AgNPs synthesis [78,79]. Some researchers have systematically investigated the green synthesis of AgNPs using a microwave-assisted approach [80,81]. The results showed that the microwave-assisted method is a fast and efficient procedure for stable AgNP synthesis from *Eucalyptus globulus* leaf extract, which had significant antibacterial activity against *E. coli* and *S. aureus* [82]. However, the question remains unsolved. Is it possible to find the most suitable way to control the size and morphology of AgNPs to boost the antimicrobial activities and biomedical applications?

## 3. Biomedical Applications of Chinese Herb-Synthesized AgNPs

China is abundant in plant resources, and the majority of its medications are derived from natural products. The development of modern drugs from natural products was mostly emphasized in the 19th century. Chinese herb-derived medicine has been primarily used as a therapeutic therapy for several decades since it had fewer side effects and complications [83]. Non-toxic Chinese herbs are a suitable candidate for the biosynthesis of AgNPs because these are readily available, active at low concentrations, and are potential reducing agents [84]. Safflower (*Carthamus tinctorius*), is a Chinese herb vastly used to treat chronic illness, dysmenorrhea, postpartum, abdominal pain, and cardiovascular complications. The phytochemistry analysis of herbal extracts revealed the presence of bioactive components, such as alkaloids, flavonoids, coumarins, fatty acids, and steroids [85,86]. *Rhodiola rosea*, also known as ‘golden roots,’ has been used for centuries to treat fatigue, anxiety, stress, and improve cardiovascular function due to the presence of salidroside and tyrosol that may increase myocardial contraction [87].

**Figure 2:**
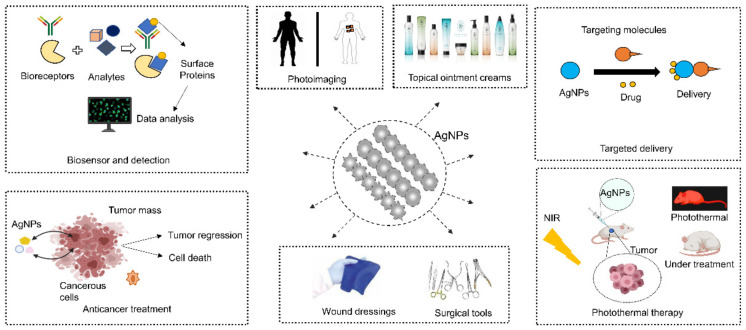
Various biomedical applications of green synthesized AgNPs.

Chinese herbs producing secondary metabolites and bioactive compounds have acquired a significant interest in biomedicine and phytonanotechnology. They act as antimicrobials, slow lipid degradation, and enhance food quality. Moreover, these phytochemicals best serve as biological drug delivery vehicles, nanosensors, and anticancer, and anti-inflammatory agents [16,88]. Therapeutic efficacy against the Ebola virus manifests the correlation with tetrandrine, an alkaloid derived from *Stephania tetrandra* [89]. Because of a large number of physiologically relevant proteins and small molecules, current research is focusing on the development of anti-rheumatoid arthritis medicine derived from Chinese herbs [90]. Furthermore, these bioactive molecules help in tissue regeneration, drug delivery, and multi-targeting and signaling pathways. A schematic illustration of the most exploited biomedical applications of biologically synthesized AgNPs in present healthcare practice is shown in Figure 2. Chinese herbal primary and secondary metabolites, including proteins, vitamins, flavonoids, polyphenols, alkaloids and polysaccharides, play a significant role in AgNP synthesis.

AgNPs are potential antimicrobial agents commonly used in clinical treatment, such as wound dressing, topical ointments and anticancer agents [91]. Recently, AgNPs with leaf and root extract of *Panax ginseng* have been successfully synthesized and showed potential antimicrobial activity. Different parts of Chinese herbs, including leaves, stenches, roots, and fruit extract, are widely applied for curing diseases. It has been reported that quasi-spherical shaped AgNPs were prepared with a size of 11.7 nm from Shanzhuyu (*Cornus officinalis*); a thin layer of water-soluble flavonoids and anthocyanins is visualized around the nanoparticles, which is responsible for their reduction and stabilization. To evaluate its anticancer activity against three cancer cell lines, it was determined that small-size AgNPs with inhibition concentration (IC_50_) values of 25.54 and 21.46 µg/mL exhibited potential cytotoxicity against human liver cancer (HepG2) and human prostate cancer (PC-3), respectively. In contrast, 50 µg/mL exhibited no cytotoxicity against human gastric carcinoma. [92]. The antioxidant properties were investigated by extracting polyphenols and anthocyanins from *Cornelian cherry* fruits to synthesize AgNPs [93]. The experiment was conducted on Wistar rats to evaluate oxidative stress parameters, apoptosis assessment and anti-inflammatory cytokine levels. Another study provided a comprehensive review on the *Cudrania tricuspidate* (Chinese mulberry), examining phytochemical constituent function in traditional use, and their antioxidant, anticancer, and anti-obesity properties [94]. Moreover, they identified and isolated phytochemicals with biological properties, including xanthones, flavonoids, organic acids, and polysaccharides. Stem, root, leaf, and fruit extracts of *C. tricuspidate* synthesized AgNPs and elicited their antibacterial, anticancer, and photocatalytic properties [95]. Barbated or baikal skullcup (*Scutellaria baicalensis*) is a natural Chinese herb containing flavonoids (Baicalin), steroids, and alkaloids that have been applied in pharmaceuticals as antimicrobial and anticancer agents [96]. AgNPs of *S. baicalensis* have been synthesized and their antimicrobial activity has been evaluated [97]. The green synthesis of AgNPs with *Cacumen platycladus* extract was identified as a reducing agent and showed potential antibacterial activity in contradiction of *E. coli* and *S. aureus* [98]. These spheroidal-shaped AgNPs were shown in a full size range of 50–100 nm, specifying that flavonoids were mainly responsible for reducing silver ions, and other active molecules ensure the protection of AgNPs. 

To correlate the size and shape of AgNPs with their bioactivity, the primary obstacle in green synthesized AgNPs is the diversity of these nanoparticles. The majority of problems encountered relate to the quality of the herbal extract, its bioactive components, the varied ratio of reagents, and reaction parameters used to control the size and stability of AgNPs [99]. The size, shape, and surface functionalization of AgNPs affect their genotoxicity and cytotoxicity. It is important to note that AgNPs smaller than 20 nm have a relatively large surface area, which ameliorates their adsorption and penetration capacity. They can easily bind to the cell membrane phosphatide, amino and carbonyl groups, and disulfide bonds in DNA that eventually cause damage to cell machinery, although some studies have outlined the effect of nanoparticle morphology on biological functions that triangular-shaped particles are more effective than spherical-shaped particles [100]. However, there is no data available on the morphology-dependent bioactivity of Chinese herbal-synthesized AgNPs. AgNPs can have a positive, neutral, or negative surface charge, depending on their synthesis technique. Abbaszadegan et al. demonstrated that altering the surface charge of nanoparticles results in a significant variation in antibacterial activity. Due to the slightly negative charge on the surface of bacteria, positively charged AgNPs are aggressively attracted to them, resulting in increased antibacterial activity. In contrast, neutral or negatively charged AgNPs have low antibacterial activity.

Previously, tremendous research has been conducted on the synthesis of AgNPs via plants and their diverse applications. However, research on Chinese herbal AgNP synthesis is still in its infancy, and few studies have been conducted on their biomedical applications due to the complex chemical composition and toxic properties. Moreover, clinical shreds of evidence and robust research validate the notion that Chinese herbs may possess broad-spectrum antimicrobial properties. A variety of Chinese herbs used for AgNPs synthesis their size, shape and biomedical applications are summarized in Table 1.

## 4. Antimicrobial Properties of Chinese Herbs

The shreds of evidence of herbal medicine in Eastern and Western cultures are about 6000 years old. Since ancient times, the use of Chinese herbs and their products has been a widespread practice to treat various diseases [56]. In recent years, phytonanotechnology, a combinational approach of Chinese herbal bioactive components, and nanotechnology have been projected as potential antimicrobial agents. Chinese herbal bioactive compounds have excellent unique surface properties, which may endorse them into an effective nano-drug delivery system. They carry potential biosensor and drug carrier properties [122]. Recently, the self-assembled small-sized phytochemicals (berberine and rhein) nanoparticles derived from TCM without nano adjuvant have shown prominent antibacterial activity against the *S. aureus* biofilm [123]. The combinational or synergistic effect of Chinese herbal AgNPs has promising therapeutic properties for targeted drug delivery systems and other extensive medical applications [124,125,126], biosensors and detection for anticancer treatment [127], and bioimaging [128]. Nowadays, AgNP-incorporated wound- healing dressings and surgical instrument coatings are clinically approved as a disinfectant because they show good antimicrobial activity. Photodynamic therapy is a new trend of non-invasive therapeutics using nanomaterials to treat cancer due to the facile and photosensitizer properties of MNPs [129,130].

### 4.1. Antibacterial Activity

Antibiotic resistance has become a major public health concern in the treatment of infectious diseases. New antibacterial agents that are resistant to bacterial targets are in high demand. Novel antibacterial agents have been discovered through the use of plant or herb-derived bioactive compounds. AgNPs synthesized from plants and herbal extract have been receiving immense interest in recent years due to their exceptional antibacterial activity and biomedical applications [131]. Among the several potential applications of AgNPs in this domain, particular emphasis and efforts have been focused on their promising implications for wound dressings, disinfectant, tissue scaffold, and protective surgical clothing. Maintaining AgNPs nanoscale size, enhancing dispersion and stability, and preventing aggregation are the key factors associated with their antibacterial activities. Many studies have shown that AgNPs have better anti-pathogenic activity than silver ions [132]. Chinese herbs are commonly used in TCM as a source of new antibacterial drugs via decoction, powder, and syrup. The antibacterial characteristics of Chinese herbs have been reported, such as *Panax ginseng* (Ginseng), *Ginkgo biloba* (Bai Guo), *Ephedra sinica* (Ma-huang), *Artemisia annua* (Qing Hao), *Alpinia officinaru* (Gao Liang Jiang), *Angelica sinensis* (Dang Gui), *Arctium lappa* (Niu Bang Zi), *Astragalus membranaceus* (Huang Qi), *Chrysanthemum morifolium* (Ju Hua), *Lycium chinense* (Chinese desert thorn), *Myristica fragrans* (Rou Dou Kou), and others [70]. *Chrysanthemum indicum* is an aromatic flowering plant, and AgNPs are prepared from flower herbal extract and screened for antibacterial effect against *B. subtilis*, *S. aureus*, *S. epidermidis*, *E. coli,* and *Pseudomonas aeruginosa* [133]. On the other hand, no cytotoxicity was observed on mice fibroblast cells, indicating that they are safe to operate. *Chrysanthemum morifolium*, a Chinese herb with a volatile flavor composition and pharmacological effects, has received much interest due to its biological characteristics such as antioxidant and anti-inflammatory properties, and work best against bacterial infections. The antibacterial activity of AgNPs synthesized by *C. morifolium* was evaluated against *S. aureus* and *E. coli* [107]. The results indicated that AgNPs at a 5–10 μg/mL concentration inhibits the growth of and kills all bacteria. To compare the bactericidal activity of AgNPs on clinical ultrasound gel, the commercial gel was contaminated with *S. aureus*, *E. coli*, *P. aeruginosa*, and *Candida albicans* (*C. albicans*), while no bacterial cross-contamination was observed on self-prepared AgNP (10 μg/mL) gel. However, the parameters and protocols used in various studies indicate that the antibacterial activity is largely dependent on the method of preparation, concentration, and particle size [73]. Additionally, the synthetic AgNPs exhibit strong antibacterial activity, which may be attributed to their small size and high surface area. 

**Figure 3:**
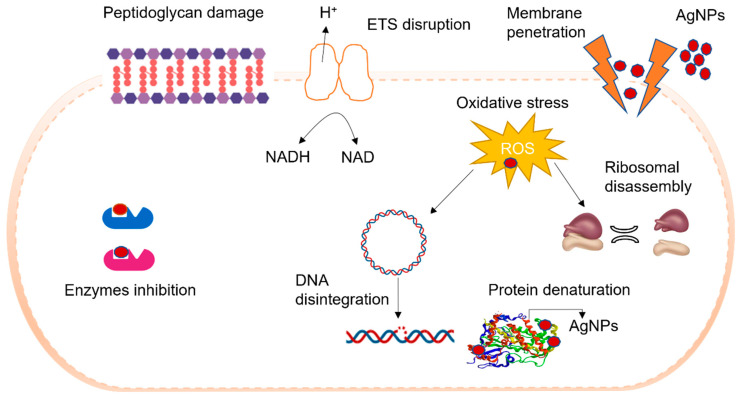
Antibacterial mechanism of Chinese herb-synthesized AgNPs. Antibacterial mechanism possibly shows that AgNPs bind to the bacterial cells and lead to the following results: (1) cell wall and cell membrane degradation, (2) penetrate intracellularly and denature proteins and damage DNA, (3) enzyme inactivation by oxidative stress generated by ROS.

AgNPs exert their intrinsic bactericidal activity against both planktonics and biofilms. The potential bactericidal mechanism of AgNPs has been explained as a Trojan-horse mechanism. Positively charged silver ions bind to the bacteria’s negative charge cell wall, inactivating cell enzymes and destroying membrane permeability. After adhesion to the bacterial surface, AgNPs can interact with cells through two different mechanisms. Small-size AgNPs (10 to 20 nm) enter directly into the cell, whereas larger nanoparticles remain outside. Interestingly, AgNPs continuously release Ag^+^ ions in both cases. These ions react with the structural protein of cell membrane, destroy the membrane potential and result in proton leakage. Cell wall instability greatly increases the permeability of bacteria, allowing larger AgNPs to enter the cells. Once they enter the cell, AgNPs and Ag^+^ ions interact with a range of structures and biomolecules, including proteins, lipids, and DNA, causing cell malfunction. AgNPs are well known for their strong capacity to generate reactive oxygen species (ROS), including hydrogen peroxide (H_2_O_2_), superoxide anions (O_2_), and hydroxyl radicals (OH). In an initial response, reactive oxygen species are produced naturally in bacteria due to cell respiration, and bacteria have defense mechanisms such as glutathione (GSH), superoxide dismutase, and catalase that serve as antioxidant enzymes and eliminate these toxic substances under normal conditions. Extreme levels of oxidative stress are caused by the high amounts of Ag^+^ produced by AgNPs. Due to their strong affinity for phosphate and carboxyl groups, these chemicals bind with respiratory chain proteins on the membrane and inactivate the enzyme [60]. Their interaction with phosphate groups inhibits protein phosphorylation, which is typically involved in enzyme activation, resulting in bacterial growth inhibition. Furthermore, the interaction of Ag^+^ with the thiol group (the functional group containing sulfur attached to a hydrogen atom) of L-cysteine results in reactive oxygen species (ROS) formation. This ROS activation leads to protein disintegration, enzyme dysfunction, and DNA damage, resulting in cell death (Figure 3) [134].

*Agrimonia herba* is a Chinese herb that contains flavonoids, phenol, and tannins. These reductive groups are required for the reduction of Ag^+^ to AgNPs via a specific mechanism. AgNPs are prepared using bioactive components have antibacterial, anticancer, and anti-inflammatory properties [109]. *Orchidantha chinensis* is a popular Chinese herb used to treat inflammatory and bacterial infections. This is the first time that AgNPs have been reported to be synthesized by an antibacterial endophyte (*Penicillium spinulosum* OC-11) isolated from *O. chinensis* and used as a reducing agent and capping agent for silver ion reduction. The disc diffusion and broth dilution assays revealed that the OC-11 strain had a strong inhibitory effect on *S. aureus*, *P. aeruginosa*, and *E. coli* [135]. *Osmanthus fragrans* grows naturally in China and is frequently used in TCM. AgNPs have been used as a reducing and stabilizing agent in combination with *O. frangrans* flower extract containing alkaloids, phenols, tannins, and flavonoids [111].

Several Chinese herbs have been investigated in recent years for their ability to produce AgNPs with significant antibacterial activity [68,89,136]. These studies demonstrate that herbal plants are readily available in nature and assure the rapid synthesis of AgNPs. Numerous studies have reported the synthesis of AgNPs from various herbal or medicinal plant extracts, including root, leaf, flower, and bark, and investigated their antibacterial activity. Chinese herbs were further investigated to treat drug-resistant bacterial infections, with 33 commonly used herbs screened for antibacterial and antiviral activity [137]. Due to the alarming rise in bacterial resistance in recent years, there is an urgent need to exploit Chinese herbal AgNPs as potential synergistic antibacterial agents.

### 4.2. Antifungal Activity

Fungal infections pose a significant threat to human healthcare systems. Fungal species have developed significant resistance to traditional and new synthetic drugs in recent years and are becoming the leading cause of death in immunocompromised patients [19]. As a result, researchers are actively involved in the development of new antifungal agents. Among biological sources, AgNPs synthesized from herbs are considered a potential source of antifungal agents due to their ability to interact with cellular contents and target virulence factors. In an Ayurvedic system of medicine, Chinese herb-based AgNPs and their role as antifungal agents have received negligible attention. It has been demonstrated that the bioactive molecules found in Chinese herbs, such as proteins, polysaccharides, and amino acids, have significant antioxidant, anticancer, anti-inflammatory, and antifungal properties [70,138,139]. The phytochemicals in the medicinal plants or Chinese herbs are receiving remarkable attention due to their potential reducing and catalytic properties. 

Previous research has reported that AgNPs have antifungal activity against pathogenic *C. albicans* at a concentration of 1 mg/mL, similar to that of commonly used antifungal agents [140]. Another study reported on the first use of *Osmanthus fragrans* leaf extract in the biosynthesis of AgNPs and analyzed and compared its antifungal activity to Tebuconazole (fungicide). AgNPs mediated by *O. fragrans* leaves had a better inhibitory effect on *Bipolaria maydis* when compared with a high efficient fungicide, Tebuconazol [141]. For optimal synthesis, several parameters that influence the synthesis of AgNPs were regulated. The inhibition effect was closely related to the concentration of AgNO_3_. To inhibit *Candida* pathogenicity, *Hypnea muciformis*, a murine macro red alga, was used to synthesize AgNPs, and their potential antifungal activity against *C. albicans*, *C. parapsilosis* and *Aspergillus niger* was evaluated [142]. The results indicated that the spherical shape and small-size nanoparticles large high surface areas bind to DNA bases and inhibit the fungi growth. The synergistic effect of *Ligustrum lucidum* leaf extract synthesized AgNPs and epoxiconazole has been investigated against *Setosphaeria turcica*, the causative agent of late blight in maize crops [143]. Maximum colony inhibition of up to 52% was observed at various AgNP concentrations (12–200 µg/mL). The size, shape, and concentration of AgNPs all affected the inhibition rate. Although the antifungal mechanism of AgNPs remains enigmatic, it has been reported that green synthesized AgNPs and their synergistic effect may be involved due to the exclusive properties of bioactive compounds as capping and reducing agents, and AgNPs bind to the plasma membrane and hamper fungal proliferation by disrupting the membrane integrity and causing structural damage [144]. After cell entry, AgNPs display a similar approach to other antimicrobials to interfere with cellular structures, hence degrading the cells.

Similarly, *Arnicae anthodium* leaf extract, which is commonly used in cosmetics, is useful for the synthesis of AgNPs. The primary bioactive components of *A. anthodium* were flavonoid and lactones, which have shown good cytotoxic activity [145]. The minimum antifungal concentration of AgNPs with an average 90 nm size was 16 µg/mL against *C. albicans*. Numerous researchers have investigated the antifungal activity of AgNPs synthesized from various medicinal herbs against *C. albicans*, working as reducing and capping agents [146,147]. Chinese herb-synthesized AgNP antifungal activities are rarely studied, possibly due to their unknown phytochemical properties. Therefore, additional research is recommended to exploit Chinese herbal medicine and its bioactive components to synthesize nanomaterials and novel antifungal agents.

### 4.3. Antiviral Properties

The recent outbreak of COVID-19 and the World Health Organization declaration of the pandemic as a public health emergency have posed a significant threat to global health and economic security. By 2020, it was critical to develop an alternative treatment to prevent and control the virus replication and spread [148,149]. It is estimated that COVID-19 has infected 194 million people, with a global death toll of 4.16 million by the end of July 15th, 2021. In the meantime, rapid and robust research is needed for effective drug development. Various evidence of Chinese herbal medicine decoctions are gradually emerging and have been recently used as a supportive treatment to boost the immune system to combat COVID-19 [150]. Herbal-based antiviral agents are supposed to be more effective and safer than synthetic drugs to prevent and treat viral infections. According to a study, the most commonly used Chinese herbal remedy for the treatment of COVID-19 is used to improve clinical symptoms, shortening the course and severity of disease and laboratory indicators [57]. A different combination of the top five Chinese herbal decoctions was employed as an antiviral agent against COVID-19, including *Radix glycyrrhizae* (Liquoric roots), *Scutellariae baicalensis* (*Baical skullcap* roots), *Pinelliae rhizoma, Forsythiae fructus,* and *Armeniacae amarum*. These herbal phytochemical constituents or polyphenols have antioxidant and antiviral effects and can be used in treating acute respiratory infections. Various Chinese herbal and their bioactive components with antiviral properties are depicted in Table 2.

Chinese herbal medicine is a rich source of secondary metabolites and bioactive compounds [69]. Recently, the stem and leaves extract of *Tinospora cordifolia*, *Phyllanthus niruri*, and *Andrographis paniculate* has been used to synthesize AgNPs, as an antiviral drug against chikungunya (a viral infection transmitted by a mosquito). The antiviral potential of these AgNPs with a size of 50–95 nm was assessed by evaluating the in vitro cell viability. The bioactive compounds in the herbal extracts contribute to the reduction of silver ions and the stability of newly formed AgNPs [158]. These findings suggest that AgNPs have been considered the best strategy as an antiviral agent to interfere and block the entry and attachment of the chikungunya virus to the host cells. 

In line with previous studies, AgNPs were synthesized from the aerial extracts of *Lampranthus coccineus* and *Malephora lutea* and their antiviral activity was studied. Based on the results, the AgNPs prepared from *L. coccineus* have effective antiviral activity against herpes simplex virus, H1N1 (influenza strain) and hepatitis B virus [159], although AgNPs interfere with viral envelope glycoproteins and prevent them from entering the host cell. Further analysis showed that AgNPs could enter the virus cells and interact with the RNA or DNA of a viral genome or through the pathways required to inhibit virus replication. However, the interaction between AgNPs and different cell types is a complex problem, so the exact mechanism of its antiviral effect is still obscure [160,161].

Phytochemicals present in herbal extracts can easily dissolve in non-polar solvents. The main components, such as alkaloids, tannins, saponins, flavonoids, or lignans are poorly soluble in water, while oral intake reduces its absorption. The essential oils also possess bioactive compounds, such as terpenoids, phenylpropanoids and alkaloids, responsible for biological activities. The ability of these oil nanocarrier systems has proved the potential antiviral activity [162]. Therefore, nanoparticles have been developed as carriers to transfer the specific biomolecules to their specific target sites [163]. In recent years, phytochemical molecule-based AgNPs are gaining more attention as they show potential antiviral activities against HIV, hepatitis B virus (HBV), and H1N1 influenza A virus. In vitro antiviral properties of AgNPs have been reported with HIV-1, HBV, and influenza virus, and the mechanism showed that AgNPs bind to one of the HIV surface glycoproteins that can influence the viral attachment to a cell. However, the details of the antiviral mechanism remain in their infancy [19]. A recent study reported that tannic acid-modified AgNPs could treat genital herpes infection, suggesting that tannic acid-derived AgNPs may be an effective antiviral drug against HSV-2 immune response. Astragali radix is the root of *Astragalus membranaceus*, and is a traditional herbal medicine used to treat kidney diseases and antiviral agents recorded in Shennong’s materia medica. Bioactive molecules of the herbal extract have a remarkable ability to fight against viral infections [59].

Many clinical trials are still ongoing, but there is no facile antiviral treatment. However, researchers are trying to find a novel antiviral cure using different approaches, such as chloroquine, a malarial drug that may potentially attenuate viral infection [164]. Another study reported the combination of TCM and Western medicines to contain COVID-19 [49]. For pandemic control, the latest development of more than 400 antiviral strategies have been implemented [165]. With the full dedication and commitment of researchers, several of these novel antiviral approaches will prove effective in treating COVID-19 disease. Hence, the biosynthesis of AgNPs from Chinese herbal medicine may likewise hold huge potential for virus prevention and control.

## 5. Further Biomedical Applications of Chinese Herb-Synthesized AgNPs

### 5.1. Antioxidant Activity

Apart from the numerous applications of AgNPs, a great number of studies have reported the antioxidant properties of green synthesized AgNPs in the last decade. In general, contradictory results can be found in previous studies on the antioxidant properties of AgNPs and extracts. Due to the occurrence of phytochemicals in the extract, they showed better scavenging activity than AgNPs. Chinese herbal medicines contain a diverse array of free radical scavenging molecules, including phenolic acid, flavonoids, terpenoids, and various other endogenous metabolites with antioxidant properties. The antioxidant activity may be attributed to the phenolic contents due to their redox properties, which allow them to act as reducing agents, hydrogen donors, and singlet oxygen quenchers. Green synthesized AgNPs as a novel therapeutic would be critical in a variety of biomedical applications [134]. AgNPs mediated by *Leptadenia reticulata* leaf extract enhanced 2,2′-diphenyl-1-picrylhydrazyl (DPPH) free radical scavenging activity. In comparison to previous studies, the highest free radical scavenging activity of AgNPs synthesized from *Leptadenia reticulate* leaf extract was 64.81% at a concentration of 500 mg/mL [166], which may be related to their ability to donate hydrogen and electron absorption in the presence of lipophilic free radicals. A similar conclusion was reached when AgNPs were synthesized from *Cibotium barometz* root extract, a Chinese herb known as “Gouji” in China [167]. AgNPs were found to exhibit strong antibacterial and antioxidant activity. To summarize, caffeic acids, protocatechuic acid (dihydroxybenzoic acid), fatty acid, and flavonoids all contribute significantly to ameliorate the antioxidant activity of AgNPs. Caffeic acid and diterpenoid extract from Saliva plants are used in folk medicines and exhibit antioxidant and anti-diabetic properties [168].

The antioxidant activity of *Elephantopus scaber* extract and AgNPs was evaluated, DPPH radical scavenging ability increased in a dose-dependent manner. The results revealed that the lowest concentration of AgNPs 50 µg/mL scavenging ability was 15.23 ± 0.04% and this antioxidant activity was improved to 85.90 ± 0.08%, when AgNP concentration increased to 250 µg/mL [169]. Previously, AgNPs synthesized from garlic, green tea, and turmeric extracts showed potential antioxidants properties. These extracts contain a high concentration of bioactive components, especially polyphenols, and act as a reducing and capping agent for AgNPs [21]. These antioxidant compounds have been reported in epidemiological studies to have anti-inflammatory, anticancer, and antibacterial or antiviral activity [170]. Another study examined the ethnopharmacology and antioxidant activity of 12 Chinese herbs [74]. *Carthamus tinctorius*, referred to as safflower in China, is a medicinal herb from which 104 compounds have been isolated and identified [85]. Because ROS are widely believed to play a role in developing numerous diseases, safflower should have a beneficial effect on cancer therapy. *Scutellaria baicalensis* (Huang-Qin) is a Chinese herb that contains flavones such as baicalin, wogonin, and aglycones. The characterization of biosynthesized AgNPs from *S. baicalensis* aqueous extract revealed its antioxidant activity against DPPH [97]. DPPH is a stable, free radical scavenging organic chemical compound useful in the screening of antioxidants. As a result, the antioxidant activity of numerous Chinese herbal and medicinal plants remains unknown.

### 5.2. Toxicity and Anticancer Activity of Chinese Herbal AgNPs

**Figure 4:**
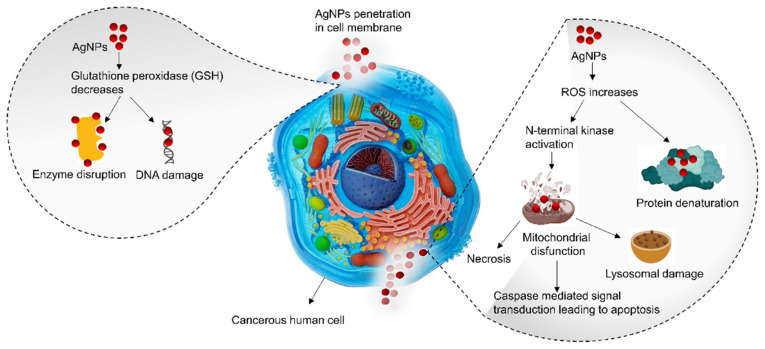
Illustration of the proposed mechanism for anticancer activity of AgNPs.

Chinese herb-derived medicine has been used as an anticancer agent and a rich source of anticancer compounds for the last two decades. Chinese herbal medicine is often preferred as a biological entity for green synthesis AgNPs that play a special role in modern anticancer treatments in vitro and in vivo. AgNPs are plasmonic structures that can scatter and absorb light impinging in certain areas, which can be used for imaging purposes. Due to their exclusive properties, AgNPs are envisioned to have great anticancer potential in two perspectives: they manifest intrinsic anticancer properties and facilitate sustained and controlled release of anticancer drugs. Currently, the theranostics approach (diagnosis and treatment) is one of the leading interests and challenging strategies for personalized anticancer therapy. Similar to the antimicrobial properties of AgNPs, anticancer activity also depends on the intracellular uptake of nanoparticles through diffusion, phagocytosis, and receptor-mediated endocytosis [171]. The cytotoxicity of AgNPs depends on the physiochemical properties, such as size, shape, and surface properties, which could deliver their internalization by cancer cells. For example, a study report that AgNPs with a diameter of 100–150 nm and a spherical shape of 30 nm asserted imminent cytotoxic effects on human lung epithelial (A-549) cells [172]. The possible explanation for high cytotoxicity may be that the small-sized nanoparticles can directly attach to the cell surfaces, release silver ions, and induce oxidative stress. These changes can cause the death of cancer cells by two basic mechanisms, apoptosis and structural and functional impairment of cellular organelles, such as protein and enzyme denaturation, mitochondrial disruption and DNA damage, as illustrated in Figure 4.

Mitochondrial-dependent apoptosis of lung cancerous cells by biosynthesized AgNPs with a 13–40 nm size in spherical shape have inferred cell cycle arrest. Moreover, the cytotoxic or anticancer effects of nanoparticles are also dose-dependent; AgNPs formulated at lower doses are considered safe. In this regard, Gomathi and co-workers recently reported that the cytotoxicity of biosynthesized AgNPs using *Tamarindus indica* leaf extract and MTT assay was carried out in different concentrations ranging from 0–120 µg/mL, and results indicated that IC_50_ at 20 µg/mL has significantly hindered the growth of human breast cancer (MCF-7) cells [173]. Similarly, an in vitro study of MCF-7 cells viability has significantly decreased AgNO_3_ at IC_50_ of 29.6 µg/mL. Likewise, Chinese herb-synthesized AgNPs with a size of 11.7 nm showed potential cytotoxicity against HepG2 at IC_50_ of 21.46 µg/mL [92]. To further consolidate the anticancer mechanism of action and scientific base of AgNPs, Chinese herbs contain a substantial amount of bioactive components with potential anticancer properties, and their synergistic AgNPs could be the best alternative for therapeutic properties.

**Figure 5:**
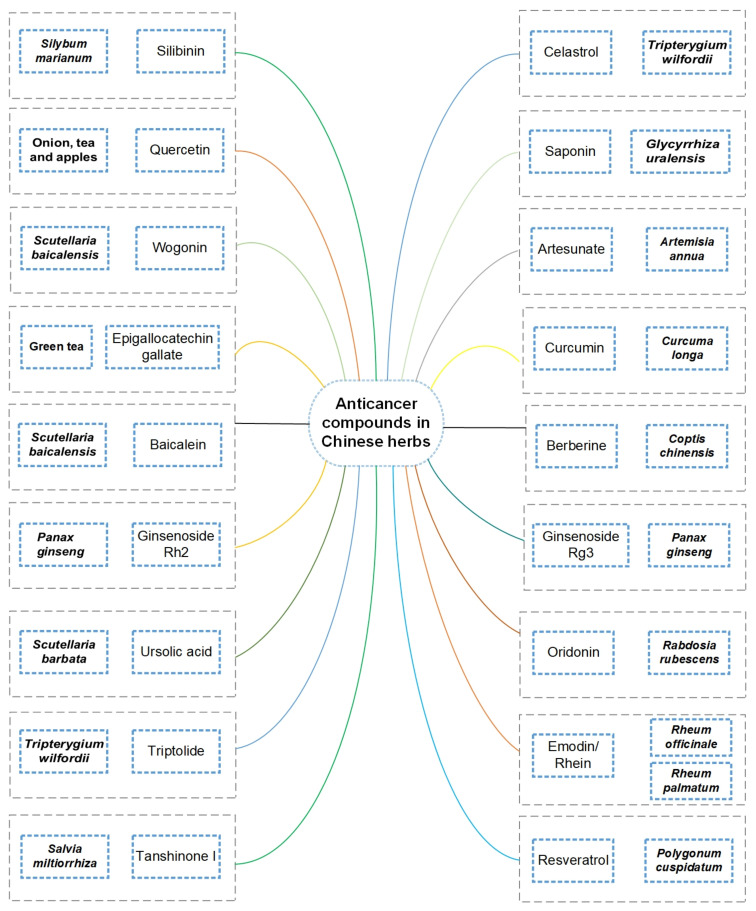
Major anticancer bioactive compounds isolated from Chinese herbs are presented.

Curcumin-induced apoptotic cell death has increased, as reported in the American Journal of Traditional Chinese Medicine. Under ultrasonic radiation, the biosynthesis of spherical AgNPs with an average size of 27.3 nm using *Sea buckthorn* berry extract indicated strong in vitro anticancer and antioxidant activity against human colorectal cancer (HCT116 and SW620), HepG2, MCF-7, and cervical cancer (HeLa) cell lines. [174]. Similarly, *Panax ginseng*-based AgNPs showed toxicity to B16 murine tumor cells but are comparatively less harmful to human dermal fibroblasts. Berberine is another natural product derived from Chinese herbs that inhibits tumor progression and is expected to be safe, efficient and affordable for cancer patients. Numerous bioactive components extracted from Chinese herbs, including curcumin, berberine, ginsenosides, silibinin, oridonin, shikonin, and cepharanthine have been reported to possess anticancer activity [175]. However, their efficiency and cellular effects are strongly dependent on the herbal bioactive components present in the extract. These compounds were identified with emerging anticancer properties, and popular compounds studied for cancer therapy are presented in Figure 5. To provide new insights, the theranostic properties of AgNPs as a drug carrier for the treatment of cancer cells have been reviewed [176]. This work proved that AgNPs can have a synergistic effect with anticancer drugs, including methotrexate, doxorubicin, alendronate, epirubicin, paclitaxel, imatinib, gemcitabine, and others. The use of a lower dose of a chemotherapeutic agent with a non-cytotoxic concentration of AgNPs has improved efficiency and reduced side effects. To date, silver has not been extensively used in drug delivery nanosystems since there are some toxicity and stability concerns. 

The in vitro anticancer activity of the Chinese herb *Cornus officinalis* (Shanzhuyu) was evaluated, along with the cytotoxic effect of Shanzhuyu-prepared AgNPs against human gastric carcinoma, prostate cancer, and liver cancer cell lines [92]. Another study reported the use of *Cibotium barometz* root extract for the synthesis of AgNPs, which acts as reducing and stabilizing agents with antimicrobial potential and cytotoxicity, in murine macrophages [167]. Four monographs on Chinese anticancer medicine have been published, and they have recorded more than 400 anticancer agents associated with Chinese herbal medicine [46]. However, the anticancer targets of these medicinal compounds are unknown, which is the major challenge in the development of Chinese herbal biomedical applications. The aqueous extract of *Oxalis corniculata* synthesized AgNPs with potential cytotoxic activity against colon cancer HT29 cells [177]. Moreover, these findings indicate that *O. corniculata* is a highly efficient reducing agent with potential biomedical applications. Licorice (roots of *Glycyrrhiza uralensis*) showed antitumor activity against a variety of cancers and has been suggested to be an effective herbal chemo-preventive medicine [178]. Many bioactive compounds present in licorice have been identified, including over 20 triterpenoids and 300 flavonoids (phenolic acid, flavones, and chalcones). Out of them all, only two triterpenoids and chalcones have shown antitumor activity. In another interesting approach, Pei et al. synthesized AgNPs in combination with aqueous leaf extract of *Coptis chineses* and studied their anticancer properties against A-549 cells [121]. They concluded that AgNPs with different concentrations (5 to 20 µg/mL) showed cytotoxic activity. Transwell assay revealed the significant inhibition of cell invasion and migration properties of A-549 cells, which is a hallmark of cancer progression. This implies that the combination of Chinese herbal medicine and silver for cancer treatment deploys the antique theory of Ayurvedic medicine. In the future, the therapeutic properties, targeted drug delivery, and clinical manifestations of Chinese herb-derived bioactive molecules will be promising areas for cancer prevention and treatment.

### 5.3. Wound Healing Properties

Wound healing is a complex biological process that occurs in response to skin injury or trauma. An intricate wound-healing process is triggered, involving a cascade of overlapping cellular and molecular interactions that eventually results in tissue recovery by restoring its defense barrier function. The wound-healing process is generally classified into four stages: hemostasis, inflammation, proliferation, and maturation [179]. Following platelet release, neutrophils migrate to the site of infection and fibrin matrix accumulation occurs. Subsequently, after two to three days of wound injury, monocytes are released and developed into macrophages for the wound-healing process [180]. Non-steroidal, anti-inflammatory drugs are widely used to treat inflammation, rheumatoid arthritis, and pain. Increased levels of proinflammatory cytokines are released by bacterial endotoxins, which inhibit growth factor synthesis and collagen deposition in wounds. Biofilm presence in chronic wounds, which is an organized consortium of bacteria encapsulated in extracellular polymeric substances produced by polysaccharides, protein, and DNA, is the most frequently encountered issue in wound enclosure due to their resistance to host immune response and antimicrobial therapies [181,182]. 

Two major types of nanoparticles are extensively exploited in wound therapy: (1) nanoparticles that carry intrinsic properties that help in wound closure; (2) they are used as vectors for the delivery of therapeutic medicines. AgNPs and their conjugates with biopolymer materials, such as collagen, gelatin, chitosan, and hyaluronic acid, have been synergistically used and accepted as generally recognized as safe [183]. They promote wound healing and inhibit bacterial growth at low concentrations to reduce cytotoxicity due to the high surface-area-to-volume ratio. AgNPs-fabricated wound dressings are a common technique for wound healing. Another promising research area is the use of glutathione as a capping and reducing agent in the synthesis of water-soluble and size-adjustable AgNPs, which play a significant role in protecting intracellular components from oxidative damage and detoxifying heavy metal ions [184]. Aside from that, the in vitro anticancer activity of AgNPs was evaluated using the human leukemia cell line K562 as a model. These findings suggest that biomolecule-capped AgNPs have a promising outlook in biomedical fields, particularly as a focal therapeutic agent for cancer therapy. To better understand the action of AgNPs in wound healing, histological sections of diabetic mice burn wounds manifest a prompt auto-inflammatory response and rapid recovery [185]. Recently, hydrogel-based AgNPs and other nanocomposite materials have been investigated for their ability to accelerate wound healing and antibacterial efficacy-enhancing properties. Animal models were used to assess the therapeutic efficacy of various hydrogels for wound healing. For instance, the release of Ag^+^ from Ag/AgCl nanocomposite hydrogels, and the therapeutic efficacy of the H_3_ and H_5_ groups in terms of wound healing, have shown cytotoxic activity [186]. *Orchidantha chinensis*, a Chinese herb, was used for the biosynthesis of AgNPs and we observed its antibacterial properties and in vivo wound healing applications. The endophytic fungus observed in *O. chinensis* attaches to AgNPs and secretes a protein that enhances antibacterial activity and wound closure using infected wound models [135]. *Aloe vera* is a medicinal plant that is mostly used in pharmaceuticals and cosmetic products. While raw leaf juice has traditionally been used as a laxative, its mucilaginous gel is generally applied to burns and cuts. Its medical significance is demonstrated by the fact that clinical studies have observed a variety of immunomodulatory properties [50].

According to our insight, thousands of plant-mediated syntheses of AgNPs and antimicrobial activities have been extensively studied, whereas Chinese herb-based AgNPs have rarely been analyzed in the previous literature as wound-healing agents. As a result, future research is needed to fully comprehend the critical tenets of Chinese herbal AgNPs for wound healing and bio-nano applications.

## 6. Conclusions and Future Prospects

In the past two decades, the green synthesis of AgNPs has gained widespread recognition due to their attractive physical, chemical, and biological properties. Despite extensive research, knowledge about the toxicity of silver is still in its infancy. Chinese herbs are a potential natural source of traditional medicine for treating various heterogeneous and complex diseases. Thus, they are widely used for pharmaceutical and clinical applications. In modern times, the Chinese traditional medicinal system, such as the concept of “yin and yang”, is not precise enough for qualitative pharmaceutical research. Researchers have explored the bioactive compounds or molecules of Chinese herbs, such as amino acids, proteins, polysaccharides, flavonoids, alkaloids, and terpenes, that hold immense potential as antimicrobial agents. However, there are few studies on the biomedical applications of Chinese herb-synthesized AgNPs. Further research is required to elucidate the phytochemical complex profile of Chinese herbs and their phenomenal therapeutic potentials and reduction properties.

Chinese herb-mediated synthesis of AgNPs is mainly studied as an antibacterial, antiviral, anticancer, and wound-healing process. At the same time, the clinical efficacy as a potential antioxidant, anticancer, and antifungal agent is worth investigating. Both AgNPs and Chinese herbs possess tremendous antimicrobial properties, and their synergistic effect will enhance their efficacy, providing a unique opportunity to address antimicrobial resistance concerns and emerging diseases, including the recent outbreak of the COVID-19 pandemic, which has now infected over 194 million people worldwide. However, the development of an efficient antiviral vaccine is underway. Apart from vaccine development, Chinese herb-mediated or TCM AgNPs may have novel therapeutic applications as an alternative approach in designing surgical equipment, gloves, and masks coated with AgNPs, which may aid in the control of and preventing COVID-19 infection. Many natural herbal products are commonly used for cancer treatment. As a result, we strongly believe that Chinese herb-based AgNPs will play a promising role in controlling COVID-19, and they are also an excellent vehicle for drug delivery to tumor cells. Yet, few clinical trials involving nanomaterials targeting cancer cells have been conducted due to the complex mechanism of targeted delivery. We highly anticipate that the Chinese herbal nano-silver approach will encourage other research groups to pursue novel biomedical applications in the future.

In short, the research progress and development of Chinese herb-synthesized silver nanoparticles, synergistic antimicrobial properties, and biomedical applications were highlighted. The bioactive molecules derived from Chinese herbs play an important role in combating antimicrobial resistance challenges and provide new avenues for pharmaceutical research to develop novel antimicrobial agents. The phytonanotechnology approach is more reliable than conventional methods to synthesize AgNPs from herbal bioactive molecules because it is a rapid, simple, low-cost technique that is non-pathogenic and offers excellent reducing and stabilizing properties. However, research on Chinese herbal phytochemicals is still ongoing and their intrinsic therapeutic properties are challenging due to their complex chemical structures and toxicity concerns. This study summarized the current development of Chinese herb-synthesized AgNPs and their synergistic use in biomedicine, such as antibacterial, antifungal, antiviral, antioxidant, and anticancer properties. This review is likely to contribute to a better understanding of the medical importance of Chinese herb-based AgNPs in the treatment and prevention of various diseases.

## Figures and Tables

**Table 1:** Chinese herbal synthesis of silver nanoparticles and their biomedical applications.

Species	Chinese Names	Parts Used or Extract	Shape	Size (nm)	Applications	References
*Cornus officinalis*	Shān zhū yú	Fruit	Quasi-spherical	11.7	Anticancer activity	[92]
*Cudrania tricuspidata*	Sāngrèn	Stem, leaf, root and fruit	Face-centered cubic crystal structure	20–50	Antibacterial, anticancer, and photocatalytic activity	[95]
*Scutellaria baicalensis*	Huáng qín	Root	Cubic	21.43	Antimicrobial, antioxidant, and anticancer activity	[97]
*Cacumen platycladi*	Cè bǎi yè	Plant	Spheroidal	18.4	Antibacterial activity	[98]
*Zingiber officinale*	Shēng jiāng	Roots	Polygonal	10	Antibacterial activity	[101]
*Gardenia* *jasminoides*	Zhī zi huā	Leaf	Spherical	10–50	Antioxidant, medical purposes	[74]
*Aloe vera*	Lú huì	Leaf	Rectangular, triangular and spherical	70	Antifungal activity	[102]
*Panax ginseng*	Rén shēn	Root	Quasi-spherical	5–15	Anticancer and antiviral activities	[103]
*Ricinus communis*	Bì má	Leaf	Spherical	8.96	Antibacterial and antimalarial	[104]
*Eclipta prostrata*	Lǐ cháng	Leaf	Spherical	45	Antimalarial	[105]
*Angelica pubescens*	Dú huó	Root	Quasi-spherical	12.48	Anti-inflammatory, analgesic, and antioxidant properties	[30]
*Astragalus* *membranaceus*	Huáng qí	Root	Spherical	65.08	Antibacterial activity	[106]
*Chrysanthemum morifolium*	Jú huā	Flower	Spherical	20–50	Antibacterial activity and clinical ultrasound gel	[107]
*Bletilla striata*	Bái jī	Tuber polysaccharides	Disc shape	5–50	Wound healing and antibacterial activity	[108]
*Agrimonia herba*	Xiān hè cǎo	Not mentioned	Spherical	11.53	Anticancer and antibacterial activity	[109]
*Chinese wolfberry*	Gǒu qǐ	Fruit	Cubic	10.9	Photocatalytic activity	[110]
*Osmanthus fragrans*	Guì huā	Flower	Spherical	20	Reducing and stabilizing agent	[111]
*Coptidis rhizome*	Huáng lián	Whole plant	Spherical	30	Antibacterial activity	[112]
*Camellia sinensis*	Chá huā	Leaf	Spherical	4.06	Cosmetics, food and medicine	[113]
*Carpesium cernuum*	*Yān gu* *ǎn t* *óu c* *ǎo*	Whole plant	Spherical	13	Antioxidant activity, and anticancer	[73]
*Ocimum basilicum*	Luó lè	Seed	Spherical	13.82	Antibacterial activity	[114]
*Rheum palmatum*	Dàhuáng	Root	Spherical and hexagonal	10–90	Antibacterial activity	[115]
*Salvia miltiorrhiza*	Dān shēn	Leaf	Spherical and hexagonal	12–80	Antibacterial and anticancer activity	[116]
*Lonicera japonica Thunb*	Jīnyínhuā	Honeysuckle extract	Not mentioned	15–17	Antimicrobial activity	[117]
*Artemisia annua*	Huánghuā hāo	Leaf	Not mentioned	1–5	Antibacterial, antioxidant and dye degradation activity	[118]
*Rehmannia glutinosa*	Shēngdì huáng	Not mentioned	Spherical	30 ± 6	Antimicrobial and catalytic activity	[119]
*Chaenomeles sinensis*	Guāng pí mù ɡu	Fruit	Cubic	5–20	Antimicrobial, antioxidant and anticancer activity	[120]
*Coptis chinensis*	Huáng liáng	Leaf	Smooth spherical	135.8	Antibacterial and anticancer	[121]

**Table 2:** Chinese herbal and bioactive compounds with antiviral effects.

Species	Bioactive Compound	Activity against	Mechanism of Action	Reference
*Lonicera* *japonica*	Chlorogenic acid, cryptochlorogenic acid, caffeic acid, luteolin, and inositol	Grouper Iridovirus	Q3-AFMP was applied to analyze the inhibitory effects of *L. japonica* components against SGIV-Gx infection	[151]
*Scutellaria* *baicalensis*	Baicalein, baicalin, wogonin, wogonoside, and oroxylin A	Influenza A virus	Increase the production of IFN-α/β and inhibit the neuraminidase activity of virus	[152,153]
*Houttuynia cordata*	Flavonoids (quercetin and isoquercetin), baicalein	Human influenza virus (H1N1)	Baicalein-triazole inhibits RSV-infection through the activation of the IFN signaling pathway	[154]
*Illicium verum*	Flavonoids, alkaloids, tri-terpenoids, saponins, tannins, and anthraquinones (Shikimic acid)	Influenza A and influenza B virus	Provide substrate for the chemical synthesis of oseltamivir phosphate	[155]
*Sambucus formosana*	phenolic acid ( caffeic acid, chlorogenic acid, and gallic acid)	Human coronavirus NL63	Inhibits RdRp	[156]
*Radix* *bupleuri*	Baicalin, puerarin, quercetin and kaempferol	SARS-CoV-2	Interact with ACE2 receptor	[157]

## Data Availability

Not applicable.
