# Correction: Islam et al. Biomedical Applications of Chinese Herb-Synthesized Silver Nanoparticles by Phytonanotechnology. *Nanomaterials* 2021, *11*, 2757

**DOI:** 10.3390/nano16080470

**Published:** 2026-04-16

**Authors:** Rehmat Islam, Leming Sun, Lianbing Zhang

**Affiliations:** Key Laboratory of Space Bioscience and Biotechnology, School of Life Sciences, Northwestern Polytechnical University, Xi’an 710072, China; rehmatislam@mail.nwpu.edu.cn (R.I.); lbzhang@nwpu.edu.cn (L.Z.)

In the original publication [1], there is a mistake in Figure 3 as published. The figure, which illustrates the antibacterial mechanism of Chinese herb-synthesized silver nanoparticles (AgNPs), incorrectly depicts a mitochondrion within the bacterial cell. As bacteria are prokaryotic organisms, they do not possess mitochondria. The corrected Figure 3 appears below.

The authors state that the scientific conclusions are unaffected. This correction was approved by the Academic Editor. The original publication has also been updated.

## Figures and Tables

**Figure 3:**
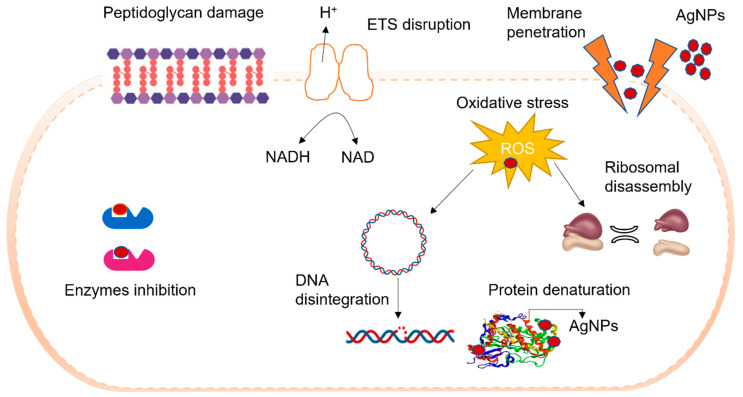
Antibacterial mechanism of Chinese herb-synthesized AgNPs. Antibacterial mechanism possibly shows that AgNPs bind to the bacterial cells and lead to the following results: (1) cell wall and cell membrane degradation, (2) penetrate intracellularly and denature proteins and damage DNA, (3) enzyme inactivation by oxidative stress generated by ROS.
